# No modifying effect of education level on the association between lifestyle behaviors and cardiovascular mortality: the Japan Collaborative Cohort Study

**DOI:** 10.1038/srep39820

**Published:** 2017-01-06

**Authors:** Eri Eguchi, Hiroyasu Iso, Kaori Honjo, Hiroshi Yatsuya, Akiko Tamakoshi

**Affiliations:** 1 Department of Public Health, Okayama University Graduate School of Medicine, Dentistry and Pharmaceutical Science, Okayama, Japan; 2 Public Health, Department of Social Medicine, Osaka University Graduate School of Medicine, Suita, Japan; 3 Osaka University Graduate School of Pharmaceutical science, Suita, Japan; 4 Department of Public Health, Fujita Health University School of Medicine, Toyoake, Japan; 5 Department of Public Health, Hokkaido University Graduate School of Medicine, Sapporo, Japan

## Abstract

We examined the effect of education level on the association between healthy lifestyle behaviors and cardiovascular mortality in the Japanese population. A total of 42,647 community-based men and women aged 40–79 years were enrolled at baseline (1988–1990), followed through 2009. The components of the healthy lifestyle score included the intake of fruits, fish, and milk; body mass index; exercise; avoidance of smoking; moderate alcohol intake; and moderate sleep duration. During the 19.3 years of follow-up, 8,314 all-cause and 2,377 total cardiovascular mortality cases were noted. Inverse associations were observed between healthy lifestyle scores and total cardiovascular disease (CVD) for both the lower and higher education level groups. Multivariable hazard ratios (95% confidence interval) for CVD mortality from the highest to the lowest healthy lifestyle scores, and the population attributable fraction (95% CIs) without healthy lifestyle scores of 7–8 were 0.51 (0.33–0.52) and 42% (24–58%), and 0.38 (0.27–0.47) and 55% (36–69%) for the higher and lower education levels, respectively. Our findings suggest that the association between higher CVD mortality and lower education level can be explained by the individuals’ lower adherence to a healthy lifestyle; hence, lifestyle modification would be beneficial for the prevention of cardiovascular mortality, irrespective of the education level.

Since the 1970s, the socioeconomic status (SES) has been well reported to affect the health and wellbeing of individuals^1^,^2^,^3^. Among the components of SES, education is considered to be one of the most powerful factors affecting the health of individuals^4^,^5^,^6^,^7^. The association between education and cardiovascular or all-cause mortality has been reported in populations worldwide^5^,^7^,^8^,^9^,^10^,^11^. For instance, in Western countries, a study of 3,456,689 person-years from 9 European countries indicated a much lower risk of cardiovascular and all-cause mortality in individuals with the highest education level, compared to those with the lowest education level^8^. In addition, in a systematic review of Asian individuals, Vathesatogkit *et al*. reported that the education level was inversely associated with cardiovascular and all-cause mortality^9^. In Japan, where the disparity in the SES is relatively small^4^,^12^, Fujino *et al*. reported that individuals with a lower education level had an increased risk of cardiovascular and all-cause mortality^5^. Another study in Japan reported that the early termination of education was associated with an increased risk of cardiovascular and all-cause mortality among both men and women^13^.

Based on these studies, it is evident that a higher education level is associated with decreased cardiovascular or all-cause mortality. However, the manner in which higher education influences the mortality risk is unclear. In the present study, we hypothesized that the deteriorated health outcomes of the lower SES population may be attributed to their poorer healthy lifestyle behaviors. Furthermore, if there was another factor mediating the effect of lower education on health outcome, the association of healthy lifestyle behaviors with such health outcomes would be different according to the education stratum.

Therefore, in the present study, we aimed to assess the effects of education level on the association between lifestyle behaviors and cardiovascular or all-cause mortality, by examining the extent of impact of lifestyle behaviors on cardiovascular disease (CVD) and all-cause mortality according to the education stratum, and by evaluating the effect modification of education on the association between lifestyle behaviors and CVD or all-cause mortality among Japanese men and women.

## Methods

- This is a large-scale, population-based cohort study of several lifestyle variables and multiple potential confounding variables.
- The impact of a combination of lifestyle behaviors according to the education level was evaluated as a risk of cardiovascular mortality.

We conducted a baseline survey of the Japan Collaborative Cohort Study (JACC study) from 1988 to 1990. A total of 110,585 subjects (46,395 men and 64,190 women) aged 40–79 years in 45 areas in Japan completed the self-administered questionnaires regarding lifestyle information and medical histories concerning CVD and cancer. The study details have been previously described in detail^14^. All the procedures were conducted in accordance with the approved guidelines. Informed consent was obtained individually from all the subjects in 36 areas (written consent in 35 areas and oral consent in 1 area), whereas written group consent was obtained from the subjects in the remaining 9 areas. This study was approved by the ethics committees of Nagoya University School of Medicine, the University of Tsukuba, and Osaka University.

Of the subjects examined, 2,574 men and 3,276 women were excluded due to a previous history of stroke, coronary heart disease (CHD), or cancer. In addition, another 12,088 men and 15,862 women were excluded due to the lack of information regarding the duration of formal education. Furthermore, another 13,291 men and 20,847 women were excluded due to the lack of information required to calculate the healthy lifestyle score. Thus, a total of 42,647 participants (18,442 men and 24,205 women) were eligible for inclusion in the present study. No major differences in the CVD risk factors were observed between individuals with satisfactory information and those with missing information.

### Mortality surveillance

The cause and date of death of participants were determined by reviewing all the death certificates from each area. According to the *International Classification of Diseases*, 10th revision, cause-specific mortality was determined in terms of stroke (I60 to I69), CHD (I20 to I25), and total CVD (I01 to I99). From the baseline until December 31, 2009, a total of 8,314 subjects were censored because of death and 2,432 subjects were censored because they moved out; in some areas, follow-up was terminated at the end of 1999 (4 areas), 2003 (4 areas), and 2008 (2 areas). The median follow-up period was 19.3 years (interquartile range, 11.6–20.8).

### Education level

Individuals with an age of **≥**16 years at the last formal education were considered as having a higher education level, whereas those with an age of <16 years at the last formal education were considered as having a lower education level. In Japan, individuals with an age of **≥**16 years at the last formal education usually finished junior high school and received high school education or higher, whereas those with an age of <16 years at the last formal education did not receive high school education or higher.

### Healthy lifestyle score (original)

The healthy lifestyle score was defined based on 8 healthy lifestyle behaviors^15^, namely: 1) fruit intake per day (**≥**1); 2) fish intake per day (**≥**1); 3) milk consumption per day (almost daily); 4) BMI (21–25 kg/m^2^); 5) number of hours spent in walking and/or sports (**≥**0.5 hours per day and/or **≥**5 hours per week, respectively); 6) avoidance of smoking (nonsmokers included past smokers); 7) alcohol consumption (<2 gou = 46.0 g ethanol/day; 1 gou is a unit of Japanese liquor that contain approximately 23 g of ethanol); and 8) sleep duration (5.5–7.4 hours/day). We assigned 1 point for each lifestyle behavior, and totaled the points to obtain the healthy lifestyle score, which ranged from 0 to 8. The scores were grouped into 5 categories (0–3, 4, 5, 6, and 7–8 points).

### Healthy lifestyle score (sensitivity analysis)

We conducted several sensitivity analyses with the modified healthy lifestyle scores. The scores included sleep duration (5.5–8.4 hours/day), as well as a binominal diet score of vegetable and bean intake in addition to fruit, fish, and milk intake. We allocated 1 point each for the diet score when the total score of fruit, fish, milk, vegetable, and bean intake was >4 points (33% of the total). Vegetable intake included the intake of spinach, garland chrysanthemum, carrot, pumpkin, tomatoes, cabbage, head lettuce, and/or Chinese cabbage, whereas bean intake included the intake of tofu, i.e. soybean curd, and/or boiled beans. Intake for more than once per day was counted as 1 point. A moderate reliability and validity for these measures have been reported elsewhere^16^.

### Measurements of covariates for multivariable analysis

To obtain the multivariable hazard ratios (HRs) of the association between lifestyle behaviors and mortality, we adjusted for the age recorded at baseline (in years) and the sex acquired by the administrative office of the JACC Study. Information on the history of hypertension and diabetes mellitus (yes or no), perceived mental stress (high, medium, or low), and regular employment (yes or no) was acquired via self-administered questionnaires. In addition to these variables, we adjusted for body mass index (sex-specific quintiles), smoking status (never, ex-smoker, current smoker of 1–19 cigarettes per day, and current smoker of **≥**20 cigarettes per day), alcohol consumption (nondrinker, ex-drinker, current drinker of 0.1–22.9, 23.0–45.9, 46.0–68.9, and **≥**69.0 g ethanol per day), hours of exercise (almost never and for 1–2, 3–4, and **≥**5 hours per week), hours of walking (almost never and for 0.5, 0.6–0.9, and **≥**1 hour per day), duration of sleeping (<4.5, 4.5–5.4, 5.5–6.4, 6.5–7.4, 7.5–8.4, 8.5–9.4, and **≥**9.5 hours), in order to obtain the multivariable HRs for the association between education level and mortality.

### Statistical analysis

We first calculated the mean age and age-adjusted prevalence of healthy lifestyle behaviors and cardiovascular risk factors according to healthy lifestyle score categories, stratified based on the education levels, using analysis of covariance. Tests for difference and linear trends were conducted using a multivariable regression model. We calculated age-adjusted and multivariable-adjusted HRs and 95% confidence intervals (CIs) to determine the sex-specific associations between education level and mortality from stroke, CHD, total CVD, and all-cause during the follow-up period by using Cox proportional hazard models. To examine the extent to which all the healthy lifestyle behaviors potentially mediated the effect of education level on CVD and all-cause mortality, we added all lifestyle scores in the model of the association between education level and CVD and all-cause mortality, and examined the magnitude of change in HRs for the highest education level, compared to the lowest education level. The proportion of CVD risk reduction explained by each set of lifestyle behaviors was calculated as [(HR original model − HR modified model)/(HR original model  − 1)] × 100%^17^.

The probability of survival from total CVD and all-cause mortality during the follow-up period for each healthy lifestyle category, stratified by education level, was calculated. Moreover, Kaplan–Meier’s survival curves were constructed to demonstrate the accumulation of mortality from baseline for each healthy lifestyle category, according to the education level. Thereafter, we estimated the multivariable-adjusted HRs and 95% CIs for mortality from stroke, CHD, total CVD, and all-cause in those with higher healthy lifestyle score categories, compared to those with the lowest lifestyle score categories (0–3) during the follow-up period, stratified by education level, by using Cox proportional hazard models. The effect modifications according to education level on the association between lifestyle behaviors and CVD and all-cause mortality were assessed using the cross-product of the education level (age at the last formal education of **≥**16 years and <16 years) and healthy lifestyle score (continuous). To investigate the combined impact of lifestyle behaviors and education on CVD and all-cause mortality, we estimated the multivariable-adjusted HRs and 95% CIs of those with higher healthy lifestyle scores and a lower education level, as well as those with higher healthy lifestyle scores and a higher education level, compared to those with the lowest healthy lifestyle scores and a lower education level as a reference category. Similarly, we also assessed the age-adjusted annual mortality rate of CVD and all-cause for each score category, based on the education level, by using the direct standardization method with the units of death per 1,000 individuals per year and with the age distribution from the national model population in 1985. Furthermore, we estimated the population attributable fraction (PAF) of not having the highest healthy lifestyle scores. The methods of PAF calculation have been described previously^15^,^18^.

In brief, equation (1).


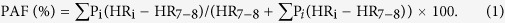


Moreover, we calculated the 95% CIs for PAF by using the method based on the Bonferonni inequality^19^. For sensitivity analysis, we estimated the multivariable-adjusted HRs and 95% CIs for total CVD and all-cause mortality with modified lifestyle scores, including sleep duration of 5.5–8.4 hours considered as a healthy lifestyle with reference scores of 0–3, as well as another sensitivity analysis with the binominal diet score (with vegetable and bean intake in addition to fruit, fish, and milk intake) in addition to the modified sleep score considered as a healthy lifestyle with reference scores of 0–1, stratified by education level, using Cox proportional hazard models. We used the Statistical Analysis Software program ver. 9.4. (SAS Institute Inc., Cary NC, USA) for all statistical analyses. All the probability values for the statistical tests were two-tailed, and P values of < 0.05 were regarded as statistically significant.

## Results

- The risk of cardiovascular mortality was reduced with an increase in the number of healthy lifestyle behaviors, irrespective of the education level.
- CVD mortality as a result of lifestyle behaviors, based on population attributable risk, was higher for lower education level than that for higher education level.

The proportion of individuals with a higher education level was 65% among the men and 63% among the women. The mean age of cases with higher education and lower education levels were 54.1 and 59.0 years, respectively, and the mean healthy lifestyle scores for cases with higher education and lower education levels were 4.3 and 4.1 among men and 5.5 and 5.2 among women, respectively. The proportion of individuals with a higher education level for the lowest lifestyle score and highest lifestyle score was 61.8% and 74.4% in men, and 49.3% and 68.9% in women, respectively. The number of deaths, during follow-up, due to stroke, CHD, total CVD, and all-cause were 530, 295, 1,240, and 4,809, respectively, among men, and 509, 197, 1,137, and 3,505, respectively, among women. Table 1 shows the sex-specific age-adjusted mean values or prevalence of healthy lifestyle behaviors and cardiovascular risk factors at baseline, based on the healthy lifestyle score categories, stratified according to the education level. Compared with individuals with the lowest healthy lifestyle score category, those with higher healthy lifestyle score categories were more likely to have higher education levels among both men and women; were more likely to be older among men; less likely to be hypertensive and more likely to have perceived mental stress among men with a higher education level; and were more likely to be younger and have a regular job among women with a lower education level.

Supplemental Table S1 shows the sex-specific age-adjusted and multivariable-adjusted HRs (95% CIs) for stroke, CHD, total CVD, and all-cause mortality according to the 3 education levels (age at last formal education of <16 years, 16–18 years, and **≥**19 years). In total, compared to individuals with the lowest education level (age at last formal education of <16 years), those with higher levels of education (age at last formal education of 16–18 years and **≥**19 years) had a lower risk of mortality from stroke (P for trend = 0.001), total CVD (P for trend = 0.0003), and all-cause (P for trend < 0.0001) in the overall population. Similar associations were observed in both men and in women. After multivariable adjustment, including healthy lifestyle behaviors, these associations appeared to be attenuated, particularly in men. The proportion of the reduction in CVD mortality explained by the lifestyle behaviors was estimated, and a large proportion (36%) of the association between education level and CVD mortality was explained by these mediators; lifestyle behaviors.

**Figure 1:**
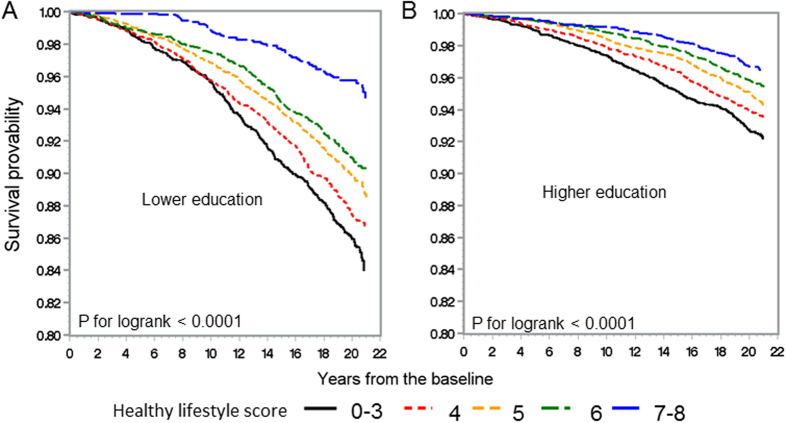
Kaplan-Meier survival curves of total cardiovascular disease mortality according to the healthy lifestyle score in the lower education level (**A**) and higher education level (**B**) groups.

**Figure 2:**
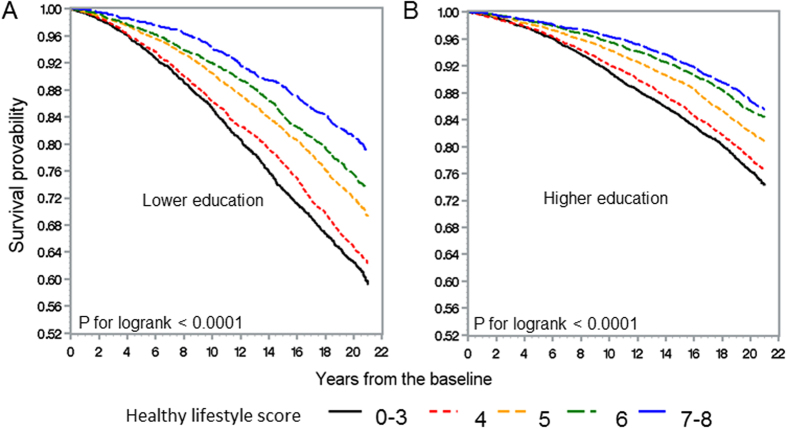
Kaplan-Meier survival curves of all-cause mortality according to the healthy lifestyle score in the lower education level (**A**) and higher education level (**B**) groups.

Figures 1 and 2 illustrate the Kaplan-Meier survival curves of total CVD and all-cause mortality according to the healthy lifestyle score in the lower and higher education level groups in the overall population. Among individuals with lower and higher education levels, those with lower healthy lifestyle score categories had worse total CVD and all-cause survival than those with higher healthy lifestyle score categories. The decline in the survival curves of the lower healthy lifestyle score categories was steeper among those with lower education level than among those with higher education level, although there were no significant interactions between the education level and healthy lifestyle score for total CVD and all-cause mortality (P for interaction: 0.23 and 0.11 for total CVD and all-cause mortality, respectively).

Table 2 shows the sex-specific multivariable-adjusted HRs (95% CIs) of stroke, CHD, total CVD, and all-cause mortality for each healthy lifestyle score category in those with higher and lower education levels. The multivariable-adjusted HRs reduced as the healthy lifestyle scores increased in a graded manner for stroke, CHD, total CVD, and all-cause mortality, among both individuals with lower and higher education levels (P for trend < 0.0001), respectively, for overall population. The same associations were observed in men and women, except for CHD mortality in men with lower education level. Although a similar trend was noted for cancer mortality and non-CVD and non-cancer mortality, the extent of risk reduction was smaller for cancer (Supplemental Table S2).

Supplemental Table S3 shows the multivariable-adjusted HR (95% CIs) of CVD and all-cause mortality for each modified healthy lifestyle score when sleep duration of 5.5–8.4 hours was considered as a healthy lifestyle behavior. The tendency was not substantially different, but the HRs of CVD mortality for those who have the highest lifestyle score in the higher education level were smaller than that of the original score. The multivariable HR was 0.41 (0.33–0.51) for the modified score, while it was 0.51 (0.33–0.52) for the original score in those with a higher education level. Supplemental Table S4 shows the multivariable-adjusted HR (95% CIs) of CVD and all-cause mortality for each modified healthy lifestyle score, with the top one-third of the binominal diet score and sleep duration of 5.5–8.4 hours considered as healthy lifestyle behaviors. Similar to the results obtained with the original score, the HRs of CVD and all-cause mortality declined gradually as the score increased. The multivariable HRs for CVD mortality among those with the highest score was 0.37 (0.27–0.50) for a higher education level and 0.32 (0.23–0.44) for a lower education level.

**Figure 3:**
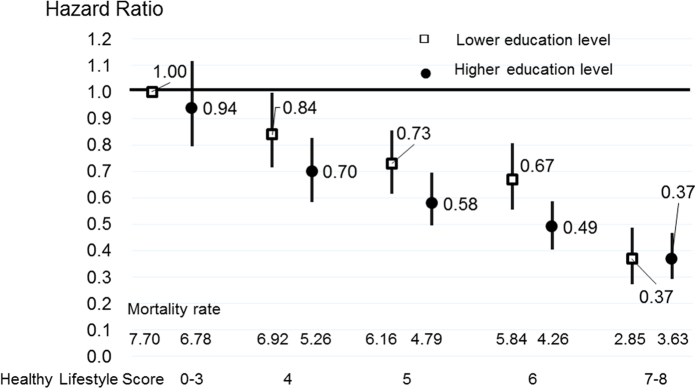
Age-adjusted mortality rate and multivariable hazard ratios (HRs) and 95% confidence intervals (CIs) for total cardiovascular mortality according to the healthy lifestyle score, stratified based on the education level for total population. Multivariable HRs were adjusted by age, sex, history of hypertension, history of diabetes, perceived mental stress and regular employment. Mortality rate were adjusted by age and sex.

**Figure 4:**
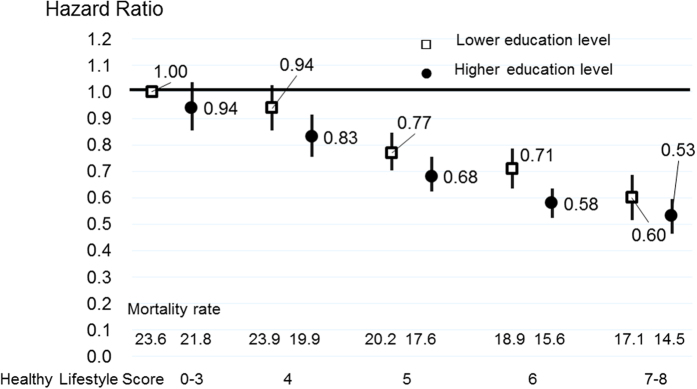
Age-adjusted mortality rate and multivariable hazard ratios (HRs) and 95% confidence intervals (CIs) for all-cause mortality according to the healthy lifestyle score, stratified based on the education level for total population. Multivariable HRs were adjusted by age, sex, history of hypertension, history of diabetes, perceived mental stress and regular employment. Mortality rate were adjusted by age and sex.

Figures 3 and 4 indicate the CVD and all-cause mortality rates for each healthy lifestyle category, stratified by education level adjusted for age and sex, as well as the multivariable-adjusted HRs (95% CIs) according to the lifestyle score categories, with the lowest healthy lifestyle score for total CVD and all-cause mortality used as a reference for the total population. Supplemental Table S5 shows the same results for men and women. The mortality rates of total CVD gradually decreased as the healthy lifestyle score increased in those with higher and lower education levels. These trends were observed in both men and women (Supplemental Table S5). The multivariable HRs of CVD and all-cause mortality for those with the highest healthy lifestyle score compared to the reference, and the mortality rates of total CVD in individuals with a higher education level did not differ markedly from those in individuals with a lower education level, when considering the same healthy lifestyle score category.

PAF estimation indicated a certain difference between the higher and lower education levels. The PAF for not being in the highest lifestyle score category for CVD mortality was larger in the lower education level than in the higher education level in the overall population. The corresponding PAFs (95% CIs) for total CVD were 42% (24–58%) in the higher education level, and 55% (36–69%) in the lower education level. In men, it was markedly higher in the lower education level than in the higher education level; the corresponding PAFs (95% CIs) were 41% (13–63%) in those with a higher education level, and 70% (38–86%) in those with a lower education level. In women, they were 37% (10–60%) and 46% (21–65%), respectively. No such major differences were observed for all-cause mortality; the corresponding PAFs in those with a higher education level and lower education level were 29% (19–38%) and 30% (18–41%) in the overall population, 35% (20–48%) and 40% (22–55%) in men, and 20% (6–34%) and 20% (5–35%) in women, respectively.

## Discussion

In this large-scale prospective study of Japanese men and women aged 40–79 years, we assessed the effect of education level on the association between healthy lifestyle behaviors and stroke, CHD, total CVD, and all-cause mortality. We observed that the risk of cardiovascular mortality reduced with an increase in the number of healthy lifestyle behaviors, irrespective of the education level, and that there was no effect modification by the education level on stroke, CHD, total CVD, and all-cause mortality. Moreover, we observed that the PAFs of lifestyle behaviors for CVD mortality, particularly among those with lower education level, were large.

To our knowledge, this is the first study to show that the strength of the association between the number of lifestyle behaviors and CVD mortality did not markedly differ between higher and lower education level groups, and that the magnitude of the PAF of not having the highest lifestyle scores for CVD mortality was larger for the lower education level group than for the higher education level group. These findings are consistent with those of previous studies, which indicated that lifestyle was an important intermediate determinant of health disorders among individuals with a lower education level^20^. In fact, several studies have reported on the association between higher education and healthier lifestyle behaviors. Townsend *et al*. showed that a lower SES is related to the likelihood of cigarette smoking^7^,^21^. In a study of 52,029 individuals in Japan, Anzai *et al*. indicated that higher education was associated with a lower body mass index (BMI) in women, and a greater amount of exercise in older individuals^22^. Another study showed that individuals with a lower education level have a greater number of lifestyle-related factors of CVD as compared to those with a higher education level^23^.

In the present study, we observed that the difference in the proportion of individuals with a higher education level between the highest lifestyle category and lowest lifestyle category was 13% in men and 19% in women in the Japanese population. Moreover, an assessment of the modification effect showed that 36% of the CVD mortality could be explained by the lifestyle behaviors reviewed. In fact, the higher proportion of individuals with healthy lifestyle behaviors among those with a higher education level may be the major reason for the lower cardiovascular mortality.

In previous studies, we assessed the differences in the impact of healthy lifestyle behaviors in terms of age categories, smoking status, and overweight status^15^,^24^. Moreover, we found that there was no major difference in the association between lifestyle behaviors and CVD mortality, after excluding early death that occurred within 5 years from the baseline, in order to assess the possibility of reverse causation^15^,^24^,^25^. In the present study, the components of healthy lifestyle score included the intake of fruit, fish, and milk; physical activity; smoking; alcohol consumption; and sleep duration. As we mentioned before, the extent of impact of each lifestyle behavior on CVD and total mortality is different. Nevertheless, for ease of application, we performed a dichotomized analysis and allocated 1 point for each health behavior. In our previous report, we indicated that the results did not markedly differ in an analysis with a weighted healthy lifestyle score^15^.

In this study, we conducted sensitivity analyses because a sleep duration of 7.5–8.4 hours was not considered to be unhealthy based on several previous reports^26^; and because vegetable and bean intake was considered to be an important part of diet that affects CVD mortality based on several previous reports^27^,^28^. In the present study, we adopted a sleep duration of 5.5–7.4 hours as a healthy lifestyle behavior, based on the findings of a previous study in Japan^29^. The results did not magnificently differ and the highest score of modified lifestyle behavior may be also effective as a healthy lifestyle behaviors.

The salient features of the study include: (1) the large-scale, population-based cohort design, including the enrollment 42,647 participants with 8,314 cases of all-cause mortality; (2) the collection of several lifestyle variables at baseline, and the adjustment for multiple potential confounding variables; and (3) the simple calculation method used for the healthy lifestyle score, which can be easily understood by the subjects. However, the present study also had certain limitations. First, the categorization of education level based on age of the last formal education may not actually reflect the length of education received by the individuals. However, >98% of those who received 16 years of education actually completed junior high school, since the proportion of cases with prolonged absence, including non-attendance at school or disease, in junior high school was 1.98% in 1991 in Japan. Second, we used mortality rather than the incidence of CVD as an endpoint. The onset of CVD could have induced lifestyle changes and consequently influenced the mortality risk in certain individuals. Moreover, the access to healthcare itself is affected by the education level^7^, which could also influence the results. Third, the baseline lifestyle data were obtained only at the initial assessment, which could lead to non-differential measurement errors and attenuation of the associations; in fact, the actual associations may be stronger. Fourth, we excluded 67,938 participants (61.4%) who consented to participate in the study, but did not have all the necessary information. However, this exclusion did not markedly affect the results, as the baseline characteristics of the excluded subjects were similar to those included in the study^15^. Fifth, we calculated 95% CIs for PAF based on the Bonferonni inequality, which made the CIs wider than using other method^19^, in fact, the actual CIs may be narrower.

In conclusion, we observed that the education level does not have any modifying effect on the association between the number of healthy lifestyle behaviors and total CVD and all-cause mortality. Moreover, the magnitude of the PAF of lifestyle behaviors is large, particularly for total CVD mortality in individuals with a lower education level. Hence, we believe that lifestyle modification would be beneficial, regardless of the education level.

## Additional Information

**How to cite this article:** Eguchi, E. *et al*. No modifying effect of education level on the association between lifestyle behaviors and cardiovascular mortality: the Japan Collaborative Cohort Study. *Sci. Rep.* **7**, 39820; doi: 10.1038/srep39820 (2017).

**Publisher's note:** Springer Nature remains neutral with regard to jurisdictional claims in published maps and institutional affiliations.

## Supplementary Material

Supplementary Table S1–S5

## Figures and Tables

**Table 1:** Mean Age and Age-adjusted Prevalence of Cardiovascular Risk Factors according to Healthy Lifestyle Score, stratified by education level.

	Healthy Lifestyle Score (points)					
	0–3	4	5	6	7–8	P for trend
**Men**						
No. at risk (%)	5992(33)	4588(25)	4107(22)	2581(14)	1174(6)	
Higher education (last formal education **≥**16 years old), %	61.8	64.5	66.5	69.7	74.4	<0.0001
** Higher education (last formal education ≥16 years old)**						
No. at risk	3779	2996	2710	1767	849	
Age, year	52.5	53.9	54.8	55.6	56.7	<0.0001
Body mass index 21-25 kg/m^2^, %	33.5	51.4	61.6	74.1	89.0	*
Fruits **≥**1/day, %	25.8	52.6	72.1	85.1	95.4	—
Fish **≥**1/day, %	19.1	32.1	44.7	57.3	79.0	—
Milk almost every day, %	16.8	37.8	52.7	71.2	86.6	—
Habitual exercise or walking, %	51.8	68.4	76.0	82.1	92.5	—
Ethanol intake <46.0 g/day, %	46.8	68.5	78.7	87.5	94.9	—
Non-smoker, %	21.1	40.0	56.1	73.7	88.7	—
Sleep 5.5–7.4 hours/day, %	32.0	49.2	58.1	68.9	88.7	—
History of hypertension, %	18.3	17.5	16.7	16.0	15.4	0.01
History of diabetes, %	6.2	6.2	6.1	5.4	5.3	0.23
High perceived mental stress, %	27.8	27.6	27.6	28.3	33.1	0.03
Regular employment, %	81.0	82.1	81.2	83.5	83.3	0.07
** Lower education (last formal education** < **16 years old)**						
No. at risk	2213	1622	1397	814	325	
Age, year	57.7	58.5	58.8	59.4	60.1	<0.0001
Body mass index 21–25 kg/m^2^, %	30.1	47.2	60.6	73.1	86.7	—
Fruits **≥**1/day, %	26.4	52.1	69.5	81.6	93.9	—
Fish **≥**1/day, %	21.9	40.1	52.4	66.3	82.6	—
Milk almost every day, %	14.5	33.6	49.0	66.2	86.5	—
Habitual exercise or walking, %	58.0	74.2	83.0	88.9	95.6	—
Ethanol intake <46.0 g/day, %	49.4	68.3	77.3	87.6	96.7	—
Non-smoker, %	21.6	41.9	56.4	73.1	89.6	—
Sleep 5.5–7.4 hours/day, %	24.6	42.7	51.9	63.2	84.7	—
History of hypertension, %	22.2	19.6	20.1	21.5	17.6	0.16
History of diabetes, %	6.3	6.1	5.8	6.3	5.4	0.64
High perceived mental stress, %	19.2	18.3	17.6	20.0	19.6	0.88
Regular employment, %	68.6	67.1	70.0	70.3	69.7	0.32
**Women**						
No. at risk (%)	1833 (8)	3999(17)	6547(27)	6888(28)	4938(20)	
Higher education (last formal education **≥**16 years old), %	49.3	56.8	61.3	66.3	68.9	<0.0001
** Higher education (last formal education ≥16 years old)**						
No. at risk	867	2234	4017	4608	3437	
Age, year	53.7	54.2	53.8	54.1	54.4	0.07
Body mass index 21–25 kg/m^2^, %	12.3	25.5	39.9	56.2	81.6	—
Fruits **≥**1/day, %	25.3	49.2	72.5	87.3	96.6	—
Fish ≥1/day, %	5.4	14.5	28.3	45.4	75.5	—
Milk almost every day, %	8.3	21.2	38.4	59.6	85.1	—
Habitual exercise or walking, %	31.4	52.1	65.9	79.9	92.4	—
Ethanol intake <46.0 g/day, %	95.7	99.1	99.6	99.8	99.9	—
Non-smoker, %	78.4	91.5	96.3	98.4	99.6	—
Sleep 5.5–7.4 hours/day, %	25.1	47.0	59.1	73.6	90.0	—
History of hypertension, %	17.4	18.6	16.5	16.7	16.0	0.15
History of diabetes, %	3.2	3.0	2.6	2.8	3.0	0.74
High perceived mental stress, %	24.6	23.6	22.4	22.3	22.8	0.31
Regular employment, %	36.4	37.7	35.5	36.5	38.8	0.06
** Lower education (last formal education** < **16 years old)**						
No. at risk	966	1765	2530	2280	1501	
Age, year	61.2	60.1	59.6	58.6	57.8	<0.0001
Body mass index 21–25 kg/m^2^, %	12.5	27.7	41.5	58.1	83.2	—
Fruits **≥**1/day, %	19.5	45.4	67.4	83.6	96.2	—
Fish **≥**1/day, %	7.7	20.1	35.5	54.3	80.4	—
Milk almost every day, %	7.5	18.5	34.6	54.4	80.5	—
Habitual exercise or walking, %	38.8	61.2	74.5	84.1	94.0	—
Ethanol intake <46.0 g/day, %	94.9	99.1	99.2	99.6	100.0	—
Non-smoker, %	80.1	92.2	96.5	98.2	100.0	—
Sleep 5.5–7.4 hours/day, %	20.5	35.8	50.8	67.7	85.0	—
History of hypertension, %	25.3	24.9	23.6	24.0	22.3	0.13
History of diabetes, %	4.1	4.6	3.8	3.6	3.2	0.12
High perceived mental stress, %	18.0	16.5	17.5	17.3	17.6	0.72
Regular employment, %	28.7	27.8	31.3	30.2	30.9	0.02

*Statistical testing was not conducted because of a component of the healthy lifestyle score.

**Table 2:** Sex-specific hazard ratios (HRs) and 95% confidence intervals (CIs) for stroke, CHD, total CVD and all-cause mortality according to the healthy lifestyle score stratified by education level.

	Healthy Lifestyle Score (points)					P for trend
	0–3	4	5	6	7–8	
**Men**						
** Higher education (last school year ≥ 16 years old)**						
** Person-years**	61951	48790	45382	29977	14268	
** Stroke**						
** **No.	99	75	52	40	19	
** **Multivariable HR (95% CI)	1.00	0.79 (0.59–1.07)	0.52 (0.37–0.72)	0.56 (0.39–0.81)	0.47 (0.29–0.77)	<0.0001
** Coronary heart disease**						
** **No.	59	45	47	15	7	
** **Multivariable HR (95% CI)	1.00	0.81 (0.55–1.20)	0.81 (0.55–1.18)	0.36 (0.20–0.64)	0.31 (0.14–0.68)	<0.0001
** Total Cardiovascular disease**						
** **No.	227	173	152	87	42	
** **Multivariable HR (95% CI)	1.00	0.80 (0.66–0.98)	0.66 (0.54–0.81)	0.53 (0.41–0.68)	0.46 (0.33–0.64)	<0.0001
** All-cause**						
** **No.	838	705	603	349	172	
** **Multivariable HR (95% CI)	1.00	0.91 (0.82–1.00)	0.74 (0.67–0.82)	0.60 (0.53–0.68)	0.55 (0.46–0.64)	<0.0001
** Lower education (last school year < 16 years old)**						
** Person-years**	33461	24458	21750	12801	5220	
** Stroke**						
** **No.	100	60	52	27	6	
** **Multivariable HR (95% CI)	1.00	0.75 (0.55–1.04)	0.68 (0.49–0.95)	0.56 (0.37–0.86)	0.27 (0.12–0.62)	<0.0001
** Coronary heart disease**						
** **No.	46	28	26	19	3	
** **Multivariable HR (95% CI)	1.00	0.80 (0.50–1.29)	0.77 (0.48–1.25)	0.88 (0.51–1.50)	0.31 (0.10–1.01)	0.10
** Total Cardiovascular disease**						
** **No.	224	137	116	70	12	
** **Multivariable HR (95% CI)	1.00	0.78 (0.63–0.96)	0.69 (0.55–0.86)	0.65 (0.50–0.86)	0.25 (0.14–0.44)	<0.0001
** All-cause**						
** **No.	778	580	443	254	87	
** **Multivariable HR (95% CI)	1.00	0.95 (0.85–1.05)	0.77 (0.68–0.86)	0.71 (0.61–0.82)	0.53 (0.42–0.66)	<0.0001
**Women**						
** Higher education (last school year ≥16 years old**						
** Person-years**	14125	37375	68240	79270	59831	
** Stroke**						
** **No.	17	36	52	53	29	
** **Multivariable HR (95% CI)	1.00	0.78 (0.44–1.39)	0.65 (0.38–1.12)	0.57 (0.33–0.98)	0.41 (0.22–0.74)	0.0008
** Coronary heart disease**						
** **No.	7	15	20	22	13	
** **Multivariable HR (95% CI)	1.00	0.79 (0.32–1.93)	0.60 (0.26–1.43)	0.57 (0.24–1.34)	0.44 (0.17–1.09)	0.048
** Total Cardiovascular disease**						
** **No.	43	81	126	131	70	
** **Multivariable HR (95% CI)	1.00	0.70 (0.48–1.01)	0.62 (0.44–0.88)	0.56 (0.40–0.79)	0.39 (0.27–0.57)	<0.0001
** All-cause**						
** **No.	133	296	439	457	317	
** **Multivariable HR (95% CI)	1.00	0.81 (0.66–1.00)	0.69 (0.57–0.84)	0.61 (0.50–0.74)	0.55 (0.45–0.67)	<0.0001
** Lower education (last school year <16 years old)**						
** Person-years**	14685	27701	41337	37782	25520	
** Stroke**						
** **No.	56	75	88	73	30	
** **Multivariable HR (95% CI)	1.00	0.75 (0.53–1.06)	0.69 (0.49–0.97)	0.67 (0.47–0.95)	0.50 (0.32–0.78)	0.003
** Coronary heart disease**						
** **No.	22	37	32	20	9	
** **Multivariable HR (95% CI)	1.00	0.92 (0.54–1.56)	0.64 (0.37–1.11)	0.47 (0.25–0.86)	0.38 (0.17–0.82)	0.0005
** Total Cardiovascular disease**						
** **No.	111	178	195	150	52	
** **Multivariable HR (95% CI)	1.00	0.90 (0.71–1.14)	0.78 (0.62–0.98)	0.70 (0.55–0.89)	0.44 (0.31–0.61)	<0.0001
** All-cause**						
** **No.	273	458	513	405	214	
** **Multivariable HR (95% CI)	1.00	0.94 (0.81–1.09)	0.80 (0.69–0.93)	0.74 (0.64–0.87)	0.67 (0.56–0.80)	<0.0001
**Total**						
** Higher education (last school year ≥16 years old)**						
** Person-years**	76076	86165	113622	109247	74100	
** Stroke**						
** **No.	116	111	104	93	48	
** **Multivariable HR (95% CI)	1.00	0.77 (0.59–1.00)	0.56 (0.43–0.74)	0.54 (0.41–0.72)	0.41 (0.29–0.59)	<0.0001
** Coronary heart disease**						
** **No.	66	60	67	37	20	
** **Multivariable HR (95% CI)	1.00	0.79 (0.56–1.13)	0.72 (0.51–1.02)	0.45 (0.30–0.68)	0.37 (0.22–0.62)	<0.0001
** Total Cardiovascular disease**						
** **No.	270	254	278	218	112	
** **Multivariable HR (95% CI)	1.00	0.76 (0.64–0.90)	0.64 (0.54–0.76)	0.55 (0.45–0.66)	0.51 (0.33–0.52)	<0.0001
** All-cause**						
** **No.	971	1001	1042	806	489	
** **Multivariable HR (95% CI)	1.00	0.88 (0.80–0.96)	0.72 (0.66–0.79)	0.61 (0.55–0.67)	0.56 (0.50–0.62)	<0.0001
** Lower education (last school year <16 years old)**						
** Person-years**	48146	52159	63086	50583	30739	
** Stroke**						
** **No.	156	135	140	100	36	
** **Multivariable HR (95% CI)	1.00	0.75 (0.59–0.95)	0.68 (0.54–0.86)	0.62 (0.48–0.81)	0.43 (0.29–0.62)	<0.0001
** Coronary heart disease**						
** **No.	68	65	58	39	12	
** **Multivariable HR (95% CI)	1.00	0.88 (0.62–1.24)	0.69 (0.48–0.99)	0.60 (0.40–0.90)	0.34 (0.18–0.64)	<0.0001
** Total Cardiovascular disease**						
** **No.	335	315	311	220	64	
** **Multivariable HR (95% CI)	1.00	0.82 (0.71–0.96)	0.71 (0.61–0.84)	0.65 (0.55–0.77)	0.36 (0.27–0.47)	<0.0001
** All-cause**						
** **No.	1051	1038	956	659	301	
** **Multivariable HR (95% CI)	1.00	0.94 (0.86–1.02)	0.77 (0.71–0.85)	0.71 (0.65–0.79)	0.60 (0.53–0.68)	<0.0001

Multivariable adjustment: age, sex (total population), history of hypertension, history of diabetes, perceived mental stress and regular employment.
