# Supplementary material for: No modifying effect of education level on the association between lifestyle behaviors and cardiovascular mortality: the Japan Collaborative Cohort Study
Source: Sci Rep. 2017 Jan 6;7:39820. doi: 10.1038/srep39820 (PMC5216353; doi:10.1038/srep39820)
Supplement: Supplementary Table S1–S5 [file srep39820-s1.pdf]

No modifying effect of education level on the association between lifestyle behaviors and cardiovascular mortality: the Japan Collaborative Cohort Study

Eri Eguchi, Hiroyasu Iso, Kaori Honjo, Hiroshi Yatsuya and Akiko Tamakoshi

Supplemental Table S1. Sex-specific hazard ratios (HRs) and 95% confidence intervals (CIs) for stroke, CHD, total CVD and all-cause mortality according to education level.

	Education levels (Age at last formal education)			
	<16	16-18	≥19	P for trend
**Men**				
**Person-years**	97690	144412	55957	
**Stroke**				
No.	245	202	83	
Age-adjusted HR (95%CI)	1.00	0.87 (0.72-1.05)	0.74 (0.58-0.95)	0.01
Multivariable HR (95%CI)	1.00	0.96 (0.79-1.16)	0.90 (0.69-1.16)	0.40
**Coronary Heart Disease**				
No.	122	120	53	
Age-adjusted HR (95%CI)	1.00	0.97 (0.75-1.25)	0.92 (0.67-1.27)	0.61
Multivariable HR (95%CI)	1.00	1.00 (0.77-1.30)	0.97 (0.70-1.36)	0.90
**Total Cardiovascular Disease**				
No.	559	474	207	
Age-adjusted HR (95%CI)	1.00	0.88 (0.78-1.00)	0.81 (0.69-0.95)	0.0042
Multivariable HR (95%CI)	1.00	0.95 (0.84-1.08)	0.93 (0.79-1.10)	0.34
**All-cause**				
No.	2142	1864	803	
Age-adjusted HR (95%CI)	1.00	0.86 (0.81-0.92)	0.80 (0.74-0.87)	<0.0001
Multivariable HR (95%CI)	1.00	0.92 (0.86-0.98)	0.89 (0.82-0.97)	0.002
**Women**				
**Person-years**	147024	213361	45480	
**Stroke**				
No.	322	151	36	
Age-adjusted HR (95%CI)	1.00	0.61 (0.50-0.74)	0.66 (0.46-0.93)	<0.0001
Multivariable HR (95%CI)	1.00	0.64 (0.52-0.78)	0.72 (0.51-1.02)	0.0002
**Coronary Heart Disease**				
No.	120	61	16	
Age-adjusted HR (95%CI)	1.00	0.65 (0.47-0.89)	0.78 (0.46-1.32)	0.03
Multivariable HR (95%CI)	1.00	0.70 (0.50-0.96)	0.90 (0.53-1.53)	0.13
**Total Cardiovascular Disease**				
No.	686	371	80	
Age-adjusted HR (95%CI)	1.00	0.71 (0.62-0.81)	0.69 (0.55-0.87)	<0.0001
Multivariable HR (95%CI)	1.00	0.75 (0.66-0.86)	0.76 (0.60-0.96)	<0.0001
**All-cause**				
No.	1863	1370	272	
Age-adjusted HR (95%CI)	1.00	0.86 (0.80-0.92)	0.79 (0.69-0.90)	<0.0001
Multivariable HR (95%CI)	1.00	0.91 (0.84-0.98)	0.85 (0.75-0.97)	0.002
**Total**				
**Person-years**	244713	357772	101437	
**Stroke**				
No.	567	353	119	
Age-adjusted HR (95%CI)	1.00	0.72 (0.63-0.83)	0.69 (0.56-0.84)	<0.0001
Multivariable HR (95%CI)	1.00	0.78 (0.68-0.90)	0.79 (0.64-0.97)	0.001
**Coronary Heart Disease**				
No.	242	181	69	
Age-adjusted HR (95%CI)	1.00	0.81 (0.67-0.99)	0.84 (0.64-1.10)	0.07
Multivariable HR (95%CI)	1.00	0.87 (0.71-1.06)	0.90 (0.68-1.19)	0.26
**Total Cardiovascular Disease**				
No.	1245	845	287	
Age-adjusted HR (95%CI)	1.00	0.78 (0.72-0.86)	0.74 (0.65-0.85)	<0.0001
Multivariable HR (95%CI)	1.00	0.84 (0.77-0.92)	0.84 (0.73-0.96)	0.0003
**All-cause**				
No.	4005	3234	1075	
Age-adjusted HR (95%CI)	1.00	0.85 (0.81-0.90)	0.79 (0.74-0.85)	<0.0001
Multivariable HR (95%CI)	1.00	0.91 (0.86-0.95)	0.87 (0.81-0.93)	<0.0001

Multivariable adjustment: age, sex (total population), body mass index, smoking status, alcohol consumption, hours of exercise, hours of walking, duration of sleeping, history of hypertension, history of diabetes, perceived mental stress and regular employment.

Supplemental Table S2. Sex-specific hazard ratios (HRs) and 95% confidence intervals (CIs) for Cancer and Non-CVD and Non-Cancer mortality according to the healthy lifestyle score stratified by education level

	Healthy Lifestyle Score (points)					
	0-3	4	5	6	7-8	P for trend
**Men**						
**Age of the last school year ≥16 years old**						
**Person-years**	61951	48790	45382	29977	14268	
**Cancer**						
No.	346	299	243	151	73	
Multivariable HR (95%CI)	1.00	0.96 (0.82-1.12)	0.76 (0.64-0.89)	0.66 (0.54-0.80)	0.60 (0.47-0.78)	<0.0001
**Non-CVD and Non-Cancer**						
No.	265	233	208	111	57	
Multivariable HR (95%CI)	1.00	0.94 (0.79-1.12)	0.79 (0.65-0.94)	0.59 (0.47-0.74)	0.55 (0.41-0.73)	<0.0001
**Age of the last school year <16 years old**						
**Person-years**	33461	24458	21750	12801	5220	
**Cancer**						
No.	289	234	169	96	36	
Multivariable HR (95%CI)	1.00	0.98 (0.69-1.39)	0.73 (0.52-1.02)	0.75 (0.54-1.05)	0.78 (0.55-1.09)	0.0002
**Non-CVD and Non-Cancer**						
No.	265	209	158	88	39	
Multivariable HR (95%CI)	1.00	0.97 (0.91-1.03)	0.90 (0.84-0.96)	0.80 (0.73-0.86)	0.79 (0.70-0.88)	0.0001
**Women**						
**Age of the last school year ≥16 years old**						
**Person-years**	14125	37375	68240	79270	59831	
**Cancer**						
No.	43	115	152	187	148	
Multivariable HR (95%CI)	1.00	0.98 (0.69-1.39)	0.73 (0.52-1.02)	0.75 (0.54-1.05)	0.78 (0.55-1.09)	0.048
**Non-CVD and Non-Cancer**						
No.	47	100	161	139	99	
Multivariable HR (95%CI)	1.00	0.77 (0.55-1.09)	0.72 (0.52-0.99)	0.52 (0.37-0.73)	0.49 (0.35-0.69)	<0.0001
**Age of the last school year <16 years old**						
**Person-years**	14685	27701	41337	37782	25520	
**Cancer**						
No.	40	119	141	117	89	
Multivariable HR (95%CI)	1.00	1.66 (1.16-2.37)	1.40 (0.99-2.00)	1.34 (0.93-1.92)	1.60 (1.10-2.33)	0.30

**Non-CVD and Non-Cancer**						
No.	122	161	177	138	73	
Multivariable HR (95%CI)	1.00	0.94 (0.87-1.01)	0.91 (0.85-0.98)	0.88 (0.81-0.95)	0.84 (0.77-0.91)	<0.0001
**Total**						
**Age of the last school year ≥16 years old**						
**Person-years**	76076	86165	113622	109247	74100	
**Cancer**						
No.	389	414	395	338	221	
Multivariable HR (95%CI)	1.00	0.85 (0.74-0.97)	0.61 (0.53-0.70)	0.53 (0.46-0.62)	0.50 (0.43-0.60)	<0.0001
**Non-CVD and Non-Cancer**						
No.	312	333	369	250	156	
Multivariable HR (95%CI)	1.00	0.78 (0.67-0.91)	0.64 (0.55-0.74)	0.44 (0.37-0.52)	0.39 (0.32-0.47)	<0.0001
**Age of the last school year <16 years old**						
**Person-years**	48146	52159	63086	50583	30739	
**Cancer**						
No.	329	353	310	213	125	
Multivariable HR (95%CI)	1.00	0.95 (0.82-1.11)	0.70 (0.60-0.82)	0.61 (0.52-0.73)	0.62 (0.50-0.76)	0.028
**Non-CVD and Non-Cancer**						
No.	387	370	335	226	112	
Multivariable HR (95%CI)	1.00	0.80 (0.69-0.92)	0.61 (0.53-0.71)	0.53 (0.45-0.63)	0.47 (0.38-0.58)	<0.0001

Multivariable adjustment: age, sex (total population), history of hypertension, history of diabetes, perceived mental stress and regular employment.

Supplemental Table S3. Sex-specific hazard ratios (HRs) and 95% confidence intervals (CIs) for CVD and all-cause mortality according to the modified healthy lifestyle score* according to healthy lifestyle score stratified by education level

	Healthy Lifestyle Score (points)					
	0-3	4	5	6	7-8	P for trend
**Total**						
**Age of the last school year ≥16 years ol**						
**Person-years**	52968	75118	111873	121006	98244	
**Total Cardiovascular Disease**						
No.	190	216	295	264	167	
Multivariable HR (95%CI)	1.00	0.73 (0.60-0.88)	0.66 (0.55-0.80)	0.55 (0.45-0.67)	0.41 (0.33-0.51)	<0.0001
**All-cause**						
No.	670	872	1075	993	699	
Multivariable HR (95%CI)	1.00	0.88 (0.79-0.97)	0.74 (0.67-0.82)	0.65 (0.58-0.72)	0.54 (0.49-0.61)	<0.0001
**Age of the last school year <16 years ol**						
**Person-years**	32195	46534	62488	60380	43115	
**Total Cardiovascular Disease**						
No.	242	275	327	263	138	
Multivariable HR (95%CI)	1.00	0.76 (0.64-0.90)	0.67 (0.57-0.80)	0.58 (0.49-0.70)	0.44 (0.36-0.55)	<0.0001
**All-cause**						
No.	751	895	1053	793	513	
Multivariable HR (95%CI)	1.00	0.85 (0.77-0.93)	0.79 (0.71-0.86)	0.65 (0.59-0.72)	0.61 (0.55-0.69)	<0.0001

Multivariable adjustment: age, sex, history of hypertension, history of diabetes, perceived mental stress and regular employment.
*sleeping duration of 5.5-8.4 hours was adopted as a healthy lifestyle behavior.

Supplemental Table S4. Sex-specific hazard ratios (HRs) and 95% confidence intervals (CIs) for CVD and all-cause mortality according to the modified healthy lifestyle score* according to healthy lifestyle score stratified by education level

	Healthy Lifestyle Score (points)					
	0-1	2	3	4	5	P for trend
**Total**						
**Age of the last school year ≥16 years ol**						
**Person-years**	16498	48407	100027	135849	99268	
**Total Cardiovascular Disease**						
No.	73	187	285	297	162	
Multivariable HR (95%CI)	1.00	0.76 (0.58-1.00)	0.56 (0.43-0.73)	0.47 (0.36-0.62)	0.37 (0.27-0.50)	<0.0001
**All-cause**						
No.	235	665	1140	1142	649	
Multivariable HR (95%CI)	1.00	0.88 (0.76-1.03)	0.74 (0.64-0.86)	0.60 (0.51-0.69)	0.47 (0.40-0.56)	<0.0001
**Age of the last school year <16 years ol**						
**Person-years**	9022	30038	59460	74288	45177	
**Total Cardiovascular Disease**						
No.	66	210	368	360	138	
Multivariable HR (95%CI)	1.00	0.68 (0.52-0.91)	0.57 (0.44-0.75)	0.49 (0.37-0.65)	0.32 (0.23-0.44)	<0.0001
**All-cause**						
No.	208	722	1153	1094	590	
Multivariable HR (95%CI)	1.00	0.89 (0.77-1.04)	0.75 (0.64-0.87)	0.65 (0.56-0.76)	0.52 (0.44-0.62)	<0.0001

Multivariable adjustment: age, sex, history of hypertension, history of diabetes, perceived mental stress and regular employment.
*Top 33% of the binominal diet score and sleeping duration of 5.5-8.4 hours were adopted as healthy lifestyle behaviors.

Supplemental Table S5. Sex-specific age-adjusted mortality rate and multivariable hazard ratios (HRs) and 95% confidence intervals (CIs) for CVD and all-cause mortality according to the healthy lifestyle score with the reference of lower edication and lowest healthy lifestyle score.

	Healthy Lifestyle Score (points)					P for trend
	0-3	4	5	6	7-8	
**Men**						
**Total Cardiovascular Disease**						
**Age of the last school year ≥16 years old**						
Age-adjusted mortality rate	7.07	6.16	5.53	4.67	4.10	
Multivariable HR (95%CI)	0.92 (0.77-1.11)	0.74 (0.61-0.90)	0.61 (0.50-0.75)	0.49 (0.38-0.63)	0.43 (0.31-0.59)	<0.0001
**Age of the last school year <16 years old**						
Age-adjusted mortality rate	8.12	6.32	6.31	6.19	2.29	
Multivariable HR (95%CI)	1.00	0.78 (0.63-0.96)	0.69 (0.55-0.86)	0.66 (0.50-0.86)	0.25 (0.14-0.44)	<0.0001
**All-cause**						
**Age of the last school year ≥16 years old**						
Age-adjusted mortality rate	25.33	24.78	21.70	18.68	17.18	
Multivariable HR (95%CI)	0.92 (0.83-1.01)	0.84 (0.76-0.93)	0.68 (0.61-0.76)	0.55 (0.49-0.63)	0.50 (0.43-0.59)	<0.0001
**Age of the last school year <16 years old**						
Age-adjusted mortality rate	28.86	27.97	24.82	23.02	20.86	
Multivariable HR (95%CI)	1.00	0.95 (0.85-1.06)	0.77 (0.68-0.86)	0.71 (0.61-0.82)	0.53 (0.42-0.66)	<0.0001
**Women**						
**Total Cardiovascular Disease**						
**Age of the last school year ≥16 years old**						
Age-adjusted mortality rate	6.52	4.46	4.14	3.90	3.21	
Multivariable HR (95%CI)	0.91 (0.64-1.30)	0.63 (0.47-0.85	0.57 (0.44-0.74)	0.52 (0.40-0.67)	0.36 (0.27-0.49)	<0.0001
**Age of the last school year <16 years old**						
Age-adjusted mortality rate	7.33	7.45	6.04	5.52	3.34	
Multivariable HR (95%CI)	1.00	0.89 (0.70-1.12)	0.77 (0.61-0.97)	0.69 (0.54-0.88)	0.43 (0.31-0.60)	<0.0001
**All-cause**						
**Age of the last school year ≥16 years old**						
Age-adjusted mortality rate	18.55	15.49	13.84	12.78	12.01	
Multivariable HR (95%CI)	1.03 (0.84-1.27)	0.84 (0.71-0.99)	0.72 (0.61-0.84)	0.63 (0.54-0.74)	0.57 (0.49-0.67)	<0.0001
**Age of the last school year <16 years old**						
Age-adjusted mortality rate	18.81	20.18	16.02	15.14	13.70	
Multivariable HR (95%CI)	1.00	0.93 (0.80-1.08)	0.80 (0.69-0.92)	0.73 (0.63-0.85)	0.65 (0.55-0.78)	<0.0001

Multivariable adjustment: age, history of hypertension, history of diabetes, perceived mental stress and regular employment.
